# “It’s Only a Model”: When Protein Structure Predictions Need Experimental Validation, the Case of the HTLV-1 Tax Protein

**DOI:** 10.3390/pathogens13030241

**Published:** 2024-03-08

**Authors:** Christophe Guillon, Xavier Robert, Patrice Gouet

**Affiliations:** Retroviruses and Structural Biochemistry Team, Molecular Microbiology and Structural Biochemistry, UMR 5086 CNRS-Lyon 1, CNRS, Université de Lyon, 69007 Lyon, France; xavier.robert@ibcp.fr (X.R.); patrice.gouet@ibcp.fr (P.G.)

**Keywords:** HTLV-1, Tax, structure, prediction, model

## Abstract

Human T-cell Leukemia Virus type 1 (HTLV-1) is a human retrovirus responsible for leukaemia in 5 to 10% of infected individuals. Among the viral proteins, Tax has been described as directly involved in virus-induced leukemogenesis. Tax is therefore an interesting therapeutic target. However, its 3D structure is still unknown and this hampers the development of drug-design-based therapeutic strategies. Several algorithms are available that can be used to predict the structure of proteins, particularly with the recent appearance of artificial intelligence (AI)-driven pipelines. Here, we review how the structure of Tax is predicted by several algorithms using distinct modelling strategies. We discuss the consequences for the understanding of Tax structure/function relationship, and more generally for the use of structure models for modular and/or flexible proteins, which are frequent in retroviruses.

## 1. Introduction

**Figure 1:**
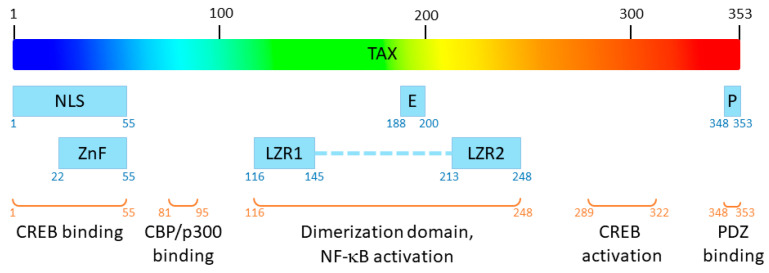
Functional domains of Tax. NLS: nuclear localization signal; E: nuclear export signal, P: PDZ-binding motif; ZnF: zinc finger; LZR1 and LZR2: leucine zipper regions 1 and 2.

Human T-Leukemia virus type 1 (HTLV-1) was the first oncogenic retrovirus discovered in humans [1]. It is estimated that 5 to 10 million people are infected with HTLV worldwide, in areas of high endemicity [2]. HTLV is the etiological agent of adult T-cell leukaemia (ATL) and Tropical Spastic Paraparesis (TSP) which occur in 5 to 10% of infected people [2]. Interestingly, only HTLV type 1 virus (HTLV-1) but not its type 2 homolog (HTLV-2) induces ATL in humans [3]. Among HTLV-1 proteins, Tax plays a central role in viral replication and HTLV-1–related pathologies [4]. Tax is a 353-residue-long viral protein (~40 kDa), in which several functional domains have been described [5] (Figure 1) which confer numerous functions to the protein.

Indeed, this viral effector recruits cellular proteins such as RNA polymerase II, CREB transcription factor and p300/CBP coactivator on the viral promotor located in the 5′ LTR of the provirus to allow efficient transcription of the HTLV-1 genome [5,6]. In addition to its function as a viral transactivator, this pleiotropic oncoprotein is able to interact directly with a large panel of cellular proteins, from transcription factors [7,8] to proteins involved in cell signalling, cell cycle or apoptotic pathways [8,9] or mRNA quality control [10], thereby playing a central role in HTLV-1 oncogenesis [4,8,11]. In particular, the expression of Tax is necessary for the proliferation of primary T-cells in ATL patients [12]. Thus, Tax represents an interesting therapeutic target for treatment against ATL, and deciphering its 3D structure would be a significant breakthrough towards the development of anti-HTLV-1 drugs. Unfortunately, the experimental solving of the 3D structure of Tax remains elusive. To date, the only published structures concerning HTLV-1 Tax are that of short peptides in complex with HLA molecules [13,14,15,16,17,18,19,20,21,22,23] or structures of the last eight residues of the C-terminal extremity of Tax, forming a PDZ-binding motif, in complex with PDZ proteins [24,25,26,27].

In the absence of experimental data on the structure of a complete Tax protein, it is tempting to consider modelling this 3D structure de novo. Until recently, the algorithms for the prediction of protein 3D structures were based on homology modelling: schematically, the algorithm will compare the sequence of the protein of interest (query sequence) with sequences of proteins for which experimental structural data are available in protein structure databases, extract its predicted secondary structures, and compare with those of the sequences of proteins that were the closest homologues in the multiple sequence alignment. Then, based on these sequence/secondary structure alignments, it models the structure of the protein of interest using the 3D scaffold of the identified model(s) and a final energy minimization step. With the emergence of artificial intelligence (AI), new structure prediction pipelines have been described. Schematically, these algorithms are based on neural networks and deep learning that are aggregating the physical and geometric constraints that are present in stretches of sequences present in published protein structures as well as global constraints to generate 3D models. These recent algorithms appear to perform with high efficacy in the yearly critical assessment of protein structure prediction (CASP, https://predictioncenter.org, accessed on 3 February 2024).

In the view of these recent developments in structure prediction, we investigated whether the 3D structure of the HTLV-1 Tax protein could be accurately modelled. Thus, we performed the structure predictions of HTLV-1 Tax proteins using eight different homology or AI-driven predictors. We then observed and compared the obtained structures in order to get a better knowledge on Tax structure and its potential use for drug design strategies, but also on the potential limitations of structure prediction.

## 2. Predicting the Structure of HTLV-1 Tax

First of all, it is worth noticing that the confidence scores for the predictions are given by distinct indexes and calculation depending on the modelling algorithm. Therefore, for each prediction, we will calculate the confidence score of all the models using a single index for clarity, i.e., the composite QMEANDisCo score [28] available via the “Structure Assessment” tool (https://swissmodel.expasy.org/assess, accessed on 29 January 2024) of the Swiss-Model server [29]. A good quality prediction is expected to have a QMEANDisCo above 0.70.

### 2.1. Predictions Using Homology Modelling

We used three different servers providing structure homology modelling methods to predict the structure of Tax: Swiss-Model [29], Phyre2 [30], and I-Tasser [31,32,33].

**Figure 2:**
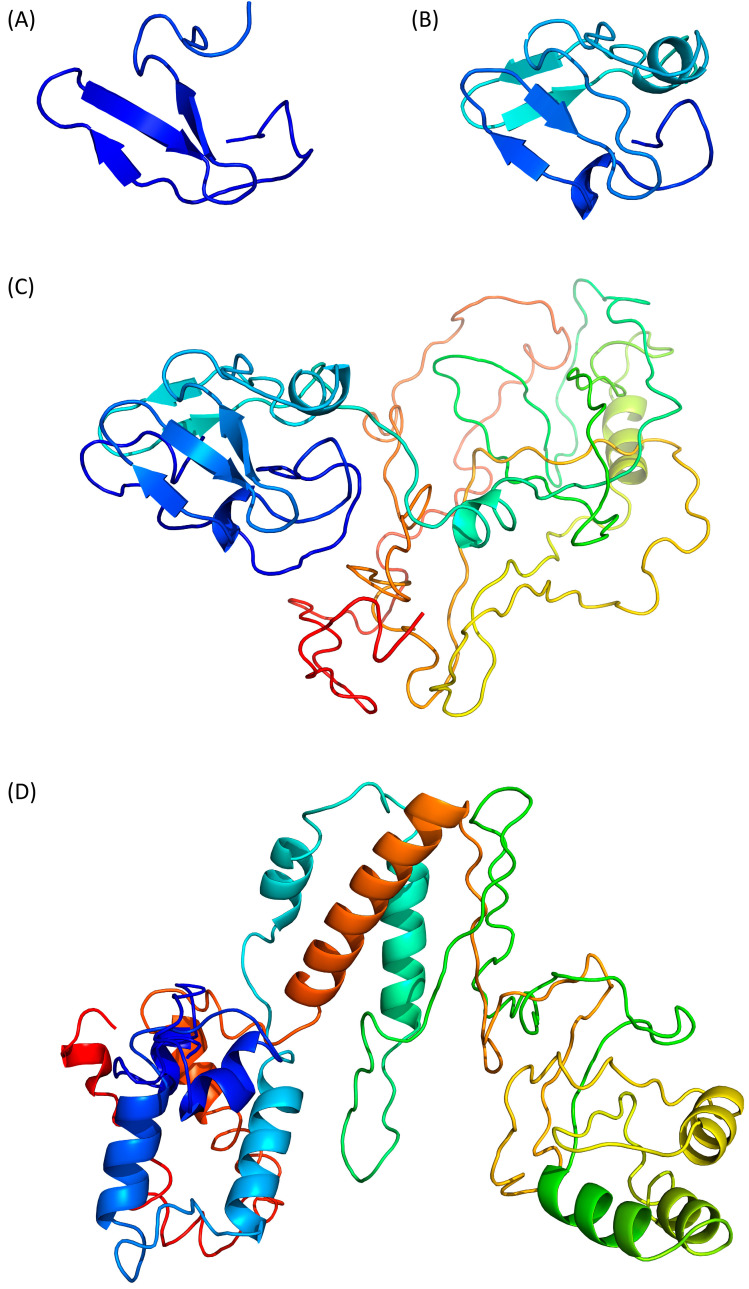
Prediction of Tax 3D structure using (**A**) Swiss-Model, (**B**) Phyre2 with defaults settings, (**C**) Phyre2 with “intensive” settings and (**D**) I-Tasser. Models are coloured from N- to C-terminal from dark blue (residue 1) to red (residue 353), as in Figure 1. Therefore, a single residue will have the same colour on all models, including the partial models (**A**,**B**).

Swiss-Model (https://swissmodel.expasy.org, accessed on 25 January 2024) predicts a β-stranded structure, which includes only 41 residues from the N-terminus of Tax (Figure 2A, residues 27–67). The predicted fragment is homologous to the nitrite reductase small subunit from *Vibrio parahaemolyticus* (PDB ID 3C0D [34]) and its confidence score QMEANDisCo is of 0.31 ± 0.12. Thus, this partial model appears poorly reliable.

Phyre2 (http://www.sbg.bio.ic.ac.uk/phyre2, accessed on 29 January 2024) predicts a structure of a 60 residue-long fragment (Figure 2B, residues 27 to 96), which encompasses the Swiss-Model structure and is homologous to the ferrodoxin component of a bacterial toluene-4-monooxygenase complex (PDB ID 1VM9 [35]). Notably, the β-strands predicted by Swiss-Model are also present and the extra modelled region contains one α-helix and two β-strands. However, the confidence score of this compact model is still low with a QMEANDisCo of 0.35 ± 0.11.

The Phyre2 server can also be used with an “intensive” option to force the modelling of the complete protein through a multiple template modelling (i.e., using several model structures based on local sequence homologies). By doing so, we obtained a model of the whole Tax protein (Figure 2C). The predicted region 27–96 is unchanged and the appended modelled parts are constructed from several other template proteins, such as a plant ferrodoxin reductase (PDB ID 1FND [36], Tax residues 207–250). The resulting predicted structure is modular with the N- and C-terminal domains separated by a flexible linker (Figure 2C) but the C-terminal part, which is not modelled with the default settings, appears to be loosely folded, with few secondary structure elements. The QMEANDisCo score of this model is lower (0.27 ± 0.05) than with the default settings. It is worth noting that this ”intensive” Phyre2 algorithm had been already used to model Tax in a publication from 2021 in which the authors reported that the model quality was also below the expected confidence scores, despite the fact that they had performed additional rounds of structure refinement [37]. 

Then, we moved to I-Tasser (https://zhanggroup.org/I-TASSER/, accessed on 26 January 2024) which is based on the assembly of PDB templates from local homology domains. The server was able to generate a structure prediction for the full protein (Figure 2D) and the first threading template was the human S-phase kinase-associated protein 2 (PDB ID 1FQV, chain A [38]). Because of this new template, the N-terminal domain of Tax is predicted to contain α-helices instead of the previous β-strands. The modelled central region (residues 100–200) and C-terminal extremity (residues 300–353) contains more secondary structure elements than the “Phyre2 intensive” model (Figure 2C,D) but the predicted tertiary structure is still loosely folded. The calculated QMEANDisCo score is also low with a value of 0.35 ± 0.05. 

In summary, the predictions of Tax structure by homology modelling give bad to mediocre results: some models are only partial (Figure 2) and all of them have a low confidence score with QMEANDisCo between 0.27 and 0.35 (Table 1). 

The variety of homology templates, together with the low confidence scores, explains the high divergence of the predicted structures, which was evident from the side-by-side comparison of the different models (Figure 2). Yet, three out of four algorithms predicted an N-terminal domain with a β-strand-rich region. This domain of Tax contains four cysteine and three histidine residues, potentially forming a zinc finger responsible for the interaction of this region with the viral DNA promoter [5]. However, none of the template proteins identified by the servers have been described to possess this motif, although they are metal-binding enzymes [34,35]. 

Altogether, these low confidence scores and the discrepancy between the templates identified by the modelling tools and what is known from Tax function confirms that, to date, no homologous structure of the Tax protein has been described. This is not completely surprising, as the sequence of Tax is the results of years of co-evolution of the virus with its host and has probably evolutionary diverged in a specific way. However, homology modelling remains an interesting approach when trying to predict the structure of a viral protein of interest, even when sequence similarity is low: there are examples of viral proteins displaying low sequence homologies (around 20%) but a similar fold because of conserved structure-function requirements (e.g., the Gag protein of lentiviruses [39]). Moreover, as the amount of protein structures available in structure databases is quickly increasing (https://www.rcsb.org/stats/growth/growth-released-structures, accessed on 2 February 2024), a HTLV-1 Tax homologue (e.g., a Tax-like protein from a distant oncoretrovirus) might sooner or later be published and therefore identified by these algorithms. Thus, it could be interesting to regularly renew these predictions as homology modelling of Tax can become conclusive.

Altogether, the use of homology modelling for Tax can only lead to the conclusion that this protein possesses a peculiar sequence for which no structural homologue could be identified to date, even for the zinc finger region. 

### 2.2. Predictions Using AI-Based Pipeline

**Figure 3:**
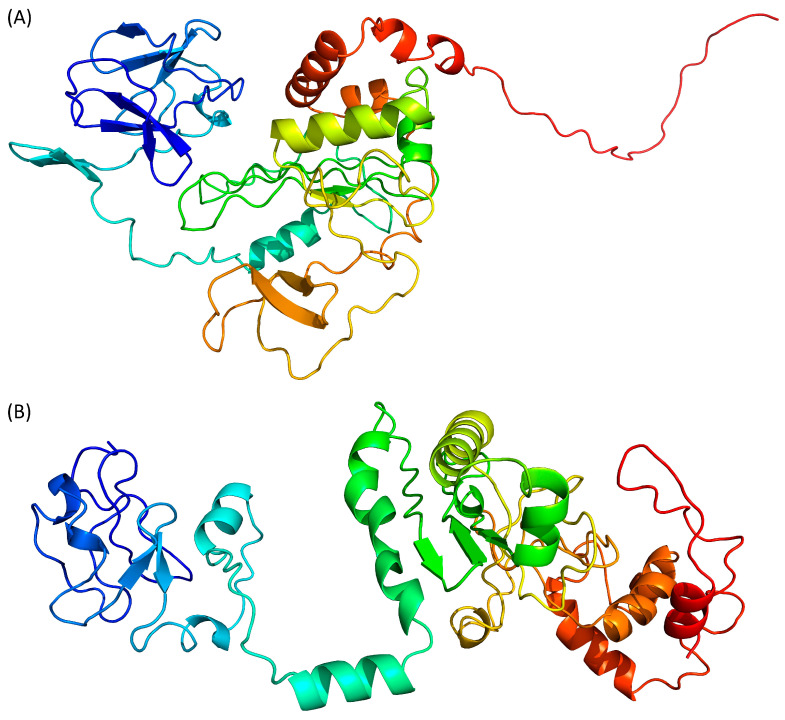

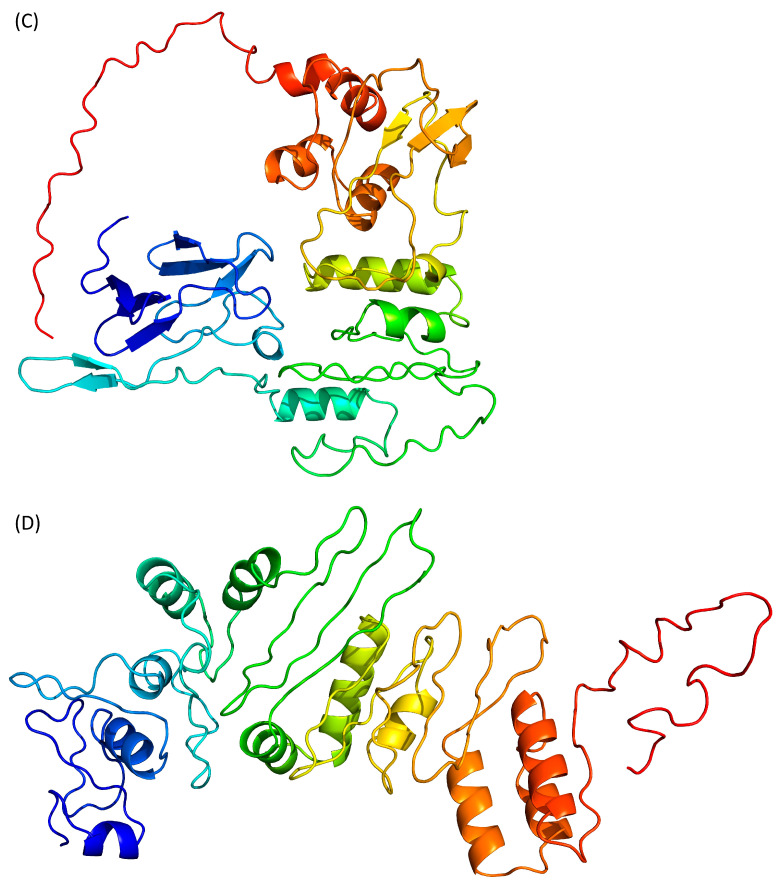
Prediction of Tax 3D structure using (**A**) AlphaFold 2, (**B**) RoseTTAFold, (**C**) ESMFold and (**D**) D-I-Tasser. Colour scheme is identical to Figure 2.

The difficulties in predicting the 3D structure of proteins that have no homologues in structure databases, as described above for Tax, is a problem which has been encountered for years. The recent appearance of AI-based algorithms, which all appeared to perform with high efficacy in international protein structure prediction competitions, has given new hopes for the deciphering ab initio of structure function relationships. Because they are based on different AI-driven processes, we have used four of them to predict the structure of Tax: AlphaFold 2 [40], RoseTTAFold [41], ESMFold [42] and D-I-Tasser [43] (Figure 3). AlphaFold 2 and ESMFold binaries were installed and run on an in-house server, while RoseTTAFold and D-I-Tasser were run through their primary webservice (https://robetta.bakerlab.org/submit.php accessed on 25 January 2024 and https://zhanggroup.org/D-I-TASSER/ accessed on 26 January 2024, respectively). The mean per-residue confidence metric called pLDDT, available in AlphaFold 2, is given for each AI-prediction when known, and the QMEANDisCo score is systematically calculated.

AlphaFold 2 is using neural networks based on evolutionary, physical and geometric constraints of protein structures [40]. The model generated by this algorithm (Figure 3A) shows a two-domains protein. The N-terminal domain is composed of β-strands while the central domain, which is rather compact, contains both β-strands and α-helices. No secondary structure elements are predicted in the C-terminal end. The confidence score pLDDT for the whole protein is 37.4, while the target values are >70 for a confident score and >90 for a very high confidence score. Even the pLDDT per residue never reaches a value above 70. This low-confidence score is confirmed by the QMEANDisCo of this model, which is 0.35 ± 0.05.

RoseTTAFold is using a “three-track network” in which information at the sequence, the secondary structure, and the 3D level are successively integrated [41]. Based on the Tax sequence, RoseTTAFold is also predicting a two-domain protein, separated by an isolated α-helix (Figure 3B). Both the N- and C-terminal domains are containing a mixture of β-strands and α-helices. However, by opposition to the AlphaFold 2 model, the C-terminal region of Tax is predicted here as containing two α-helices. The N-terminal region (residues 20–74) is predicted to contain helical motifs that are absent from the AlphaFold 2 model (Figure 3A). It is also the only region of the protein where the error estimates per residue of the model are below 6 Å. However, the overall confidence score of the RoseTTAFold model is 0.35 (target > 0.70), while a calculated QMEANDisCo of 0.39 ± 0.05. Thus, even with some locally favourable confidence scores for the N-terminal region, this model is not estimated as very reliable overall.

ESMFold adopts a different approach, as it uses language models trained on protein sequences and therefore does not depend on multiple sequence alignments [42]. ESMFold, like the other AI-based algorithms, predicts that the Tax protein is composed of two domains (Figure 3C). The N-terminal region is composed only of β-strands, while the central domain contains both β-strands and α-helices, and the C-terminal extremity is predicted as disordered. The mean pLDDT is 47.6 (target > 70) and the calculated QMEANDisCo value is 0.43 ± 0.05 (target > 0.70). Of interest, pLDDT scores per residue are between 70 and 80 (i.e., scoring as “confident”) for the residues at the N-terminus (residues 15–75).

Finally, we used D-I-Tasser which is an evolution of I-Tasser (see above) that includes a deep neural-network predictors analysis coupled to the I-Tasser force fields (Figure 3D). D-I-Tasser predicted a model for the whole protein and the first threading template is, this time, a protein from the drosophila apoptosome (PDB ID 1VT4 [44]). As a consequence, the predicted topology is different from the I-Tasser one and the D-I-Tasser model has more α-helices (Figure 2D and Figure 3D). The confidence score of the D-I-Tasser model is better than the one of I-Tasser with a QMEANDisCo of 0.44 ± 0.05, which is within the range of the other AI-programs. Yet, it remains well below the confidence threshold of 0.70.

In summary, the predictions of the structure of the whole Tax protein by AI-based modelling algorithms gave low confidence scores (QMEANDisCo between 0.35 and 0.44, Table 2), with D-I-Tasser having the best score. 

When comparing QMEANDisCo values for all the generated model (Table 1 and Table 2), it is noticeable that no algorithm performed significantly better than the others, and that both homology and AI-based algorithms reach similar low QMEANDisCo confidence scores. This means that the sequence of the Tax protein seems resistant to modelling, whether by homology or ab initio.

## 3. Comparison of HTLV-1 Tax Structure Models

**Figure 4:**
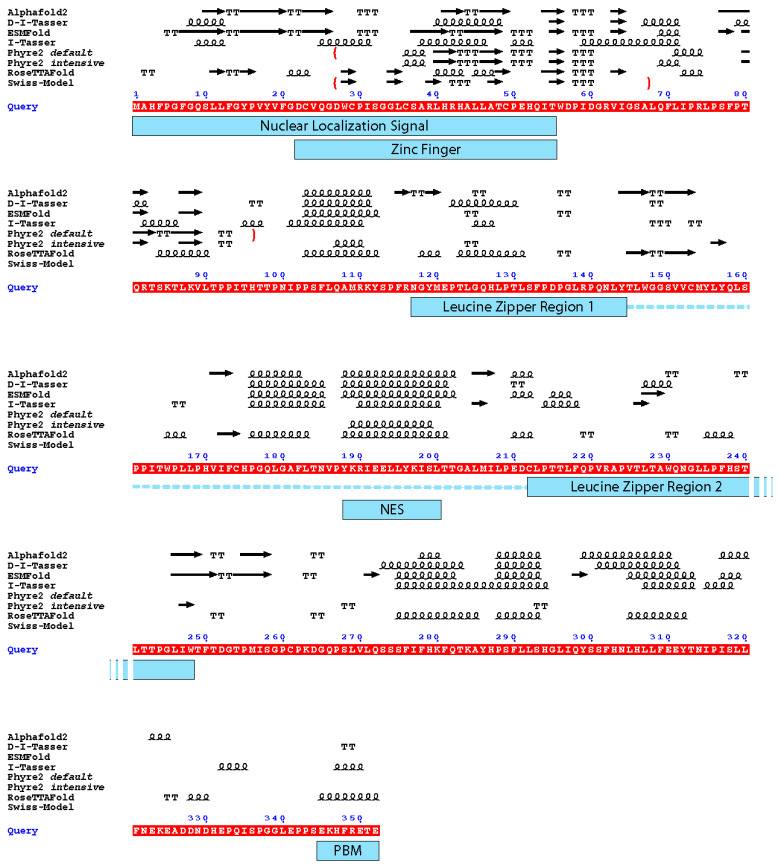
Depiction of the secondary structure elements from the different models with the functional domains of the Tax protein. Underlined in red is the sequence of Tax used for the modelling (Query). Above the sequence: arrow: β-strand. Squiggles: α-helix. T: turn. The boundaries of the partial models are depicted by red brackets. Under the sequence, blue rectangles mark the functional domains of Tax depicted in Figure 1; the dotted line together with the leucine zipper regions depict the dimerization domain of Tax. NES: Nuclear Export Signal; PBM: PDZ-binding motif. The figure was generated by ESPript 3.0 [45].

Two of the four AI-generated models (RoseTTAFold and ESMFold) exhibited the best local confidence scores for the N-terminal domain of Tax, which is the zinc finger domain which was also modelled by Swiss-Model and Phyre2. Therefore, we wondered if there could be some conserved local folding which would be identifiable although the confidence scores of the whole models were not good. Thus, we compared the secondary structures elements of all these models with respect to Tax functional regions (Figure 4).

**Figure 5:**
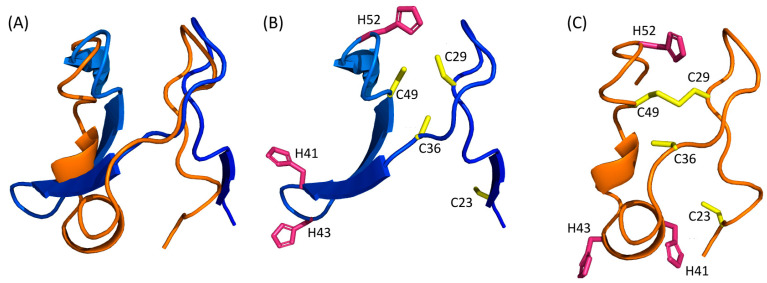
(**A**) Superimposition of the N-terminal domain (residues 15–74) of RoseTTAFold and ESMFold models. ESMFold and RoseTTAFold models are coloured with the same colour scheme as Figure 3 and orange, respectively. (**B**,**C**) CCCH zinc fingers predicted by ESMFold (**B**) and RoseTTAFold (**C**). All the cysteine residues are in yellow and histidine residues in pink.

It appears that the Nuclear Export Signal and the centre of the dimerization domain are predicted as being in an α-helical region by all predictors that modelled this region (residues 175–205). Notably, it is the region which had the best local confidence score in the D-I-Tasser model. For the rest of the protein, none of the models are convergent (Figure 4). For example, if we consider the zinc finger domain of Tax, we can observe that five models are predicted to have a β-stranded fold (AlphaFold 2, ESMFold, Swiss-Model and both Phyre2 models), while the other three (RoseTTAFold, I-Tasser and D-I-Tasser) predicted the presence of helical elements and turns, together with β-strands or replacing them. Furthermore, despite having good local confidence scores for this domain, the Cα trace of the ESMFold and RoseTTAFold models do not superpose (Figure 5A).

As there are a lot of different topologies for zinc fingers that have already been described in the literature [45], this observation could suggest that the Tax protein harbours another, yet undescribed, zinc finger topology that the algorithms do not identify, especially as they do not support the prediction of metal coordination. Indeed, although not superposing, both ESMFold and RoseTTAFold predicted three cysteines (C29, C36 and C49) and one histidine (H52) in close vicinity, which could coordinate a zinc ion (Figure 5B,C). Such zinc fingers with three cysteines and one histidine (CCCH) have been described and are involved in RNA metabolism [46]. Their consensus sequence is C-(X_4–15_)-C-(X_4–6_)-C-(X_3–4_)-H (with X for any amino-acid) [47]. Thus, this putative CCCH zinc finger in Tax, with the sequence C-X_6_-C-X_12_-C-X_2_-H, would be non-canonical and marked by a particularly longer distance between the second and third cysteines (12 instead of 4 to 6). Of note, this zinc finger is also predicted by AlphaFold 2 but not by D-I-Tasser, nor by any other homology modelling method. 

Another possibility is that this region of Tax is intrinsically disordered and that the zinc finger is only forming through induced folding when Tax interacts with a biological partner. The formation of the zinc finger of Tax could also require trans-complementation with domains or residues of the interacting partner, as it contains only seven cysteines or histidine residues while eight are needed to complete two zinc fingers. Such an induced folding and trans-complementation for the formation of the zinc finger have been described for the HIV-1 Tat protein: this regulatory protein, which is intrinsically disordered [48,49], contains seven cysteine residues and uses a residue from its interacting partner, Cyclin T1, to complete its two zinc fingers that are then folded as α-helices [50]. 

The experimental elucidation of the 3D structure of this region of Tax, alone or in complex with one of its biological partners, will be necessary to conclude on this matter.

## 4. Conclusions and Perspectives

In conclusion, the only convergent result that can be obtained from the comparisons of all these models is that the Tax protein seems to be a modular protein, containing two more or less compact domains separated by a flexible linker, with a nuclear export domain probably α-helical, and with a C-terminal end which is loosely structured and/or can adopt different folding. As soon as we try to go deeper in the details, the different models that we have obtained are divergent. This could be expected by the fact that none of the models had good confidence scores, suggesting that they are all (at least partially) wrong.

When we focus on specific functional domains such as the zinc finger, some models seemed to converge, but there are still some discrepancies, even between models that predicted this region with good confidence scores. Thus, to date, it appears that it is not possible to model the structure of the Tax protein with a sufficient accuracy to use any of these predictions to understand structure-function relationships of Tax and even less to guide a structure-based drug design. 

Notably, Tax can undergo several post-translational modifications, such as phosphorylation [51], acetylation [52], SUMOylation and/or poly-ubiquitination [53] which are important for its function [8] and may influence its conformation, as described for other proteins [54,55,56]. However, there is no algorithm to date which includes this parameter during protein structure prediction.

This work on the Tax protein has three consequences for the understanding of Tax structure, but also beyond the HTLV-1 field for the use of structure models of modular proteins. 

First, it underlies that, although the confidence scores must be the first criterion to consider, a good confidence score, even at a local scale, is not enough to discern “good” from “bad” models, as exemplified by the comparison of the N-terminal regions that showed good local confidence scores with RoseTTAFold and ESMFold (Figure 5). Therefore, even for models which are predicted with pLDDT confidence scores around 70–80%, it should be recommended to use two or three distinct structure prediction algorithms to check if there is some convergence or not. 

Second, one should keep in mind that it may difficult to predict the structure of some proteins because of their intrinsic complexity. This is particularly true for retroviral proteins such as Tax of HTLV-1. Indeed, the genome of retroviruses is about 10kb in size, but must still sustain a complete viral replication cycle. This means that retroviral proteins have often several functions (and it is the case of Tax), which force them to adopt different conformations to adapt to different needs of the virus. As a consequence, retroviral proteins tend to be modular proteins with flexible regions, that are undergoing conformational changes with reorganization of the respective orientations of some domains/subdomains [57,58]. Some of them can even be mostly intrinsically disordered and/or undergo induced folding, i.e., appearance of secondary/tertiary structure elements only in certain conditions [48,59]. Predicting such a fluctuating landscape, even at a local scale, could be unreachable for 3D modelling algorithms. This has been demonstrated for AlphaFold 2 [60,61], but it is probably a problem for all structure modelling strategies.

This leads to the third consequence of this work: algorithms predicting the structure of proteins must still be fed by experimental structural data, in order to increase their panel of possible conformations and thereby their accuracy. The case studied here clearly shows that whatever programs are used, even the most recent and innovative ones based on AI, they all find themselves faced with a “gray zone”, which does not allow them to deliver reliable predictions. It is when the structure of Tax will be experimentally solved that we will understand which model, if any, was the closest to reality and where it was wrong. It will also help identify which domains of Tax had specific, unpredictable structures. These unique features would be the best targets for the development of anti HTLV-drugs.

## Figures and Tables

**Table 1:** Summary of confidence scores for each model of Tax generated by homology modelling. QMEANDisCo scores were calculated as explained in the text.

Modelling Server	Complete Protein Modelled?	Calculated QMEANDisCo
Swiss-Model	No	0.31 ± 0.12
Phyre2 (default)	No	0.35 ± 0.11
Phyre2 (intensive)	Yes	0.27 ± 0.05
I-Tasser	Yes	0.35 ± 0.05

**Table 2:** Summary of confidence scores for each model of Tax generated by AI-based algorithms. QMEANDisCo scores were calculated as explained in the text.

Modelling Program	Complete Protein Modelled?	Original Confidence Score	Calculated QMEANDisCo
AlphaFold 2	Yes	pLDDT = 37.4	0.35 ± 0.05
RoseTTAFold	Yes	Predicted GDT = 0.35	0.39 ± 0.05
ESMFold	Yes	pLDDT = 47.6	0.43 ± 0.05
D-I-Tasser	Yes	eTM = 0.4	0.44 ± 0.05

## Data Availability

The raw data supporting the conclusions of this article will be made available by the authors on request.
